# Kaempferol Addition Increases the Antimalarial Activity of Artesunate in Experimental Mice

**DOI:** 10.1155/2020/6165928

**Published:** 2020-06-29

**Authors:** Sakaewan Ounjaijean, Nattida Benjasak, Suchanan Sae-lao, Voravuth Somsak

**Affiliations:** ^1^Research Institute for Health Sciences, Chiang Mai University, Chiang Mai 50200, Thailand; ^2^School of Allied Health Sciences, Walailak University, Nakhon Si Thammarat 80161, Thailand

## Abstract

Kaempferol (KMF) is a member of flavonol widely found in tea, broccoli, apples, strawberries, and beans. It has been demonstrated to present several pharmacological properties with potent antimalarial activity against *Plasmodium berghei*-infected mice. Hence, the search for a safe and new antimalarial compound with combinations to delay the development of resistance was the aim of this study. Thus, the therapeutic effect of the combination of KMF and artesunate (ART) in *P. berghei*-infected mice was evaluated. Combination of KMF and ART in *P. berghei* ANKA- (PbANKA-) infected ICR mice in a fixed-ratio combination (1 : 1) and fractions of their median effective dose (ED_50_) was also investigated using the standard 4-day suppressive test. The ED_50_ levels of KMF and ART in mice infected with PbANKA were 20.06 ± 2.65 and 6.06 ± 1.33 mg/kg, respectively. Moreover, KMF showed promising synergistic combination with ART at the doses of their ED_50_ and fixed-ratio combination (1 : 1) of their ED_50_ of 1/2 with combination index (CI) values of 0.86 and 0.47, respectively. Additionally, KMF, ART, and its combination at the doses of their ED_50_ and fixed-ratio combination (1 : 1) of their ED_50_ of 1/2 also presented significantly (*P* < 0.001) prolonged mean survival time (MST). The findings of this study showed that a combination of KMF and ART enhanced the antimalarial activity of ART and prolonged MST. This study supports the basis for the selection of KMF as a prospective compound for further consideration as a partner drug for ART.

## 1. Introduction

Malaria remains one of the leading causes of mortality and morbidity accounting for about 10% of childhood deaths in sub-Saharan Africa [1]. In 2018, it was estimated that 228 million cases of malaria and 405,000 malaria-related deaths occurred in the world [2]. The resistance of *Plasmodium* parasites to available antimalarial drugs especially to artemisinin and the delayed parasite clearance with the artemisinin-based combination therapy (ACT) has led to increase treatment failure [3–6]. Hence, the search for safe and selective chemotherapeutic drugs with efficacy that will not be compromised by *Plasmodium* remains the focus.

Kaempferol, a flavonol widely found in different vegetables (tea, broccoli, apples, strawberries, and beans), is known to be one of the most active and important natural compounds with several pharmacological properties including antioxidant, anti-inflammatory, antimicrobial, and anticancer [7–9]. Moreover, kaempferol has been studied and found to be an important compound in the search for an effective antimalarial drug against *Plasmodium falciparum* and *Plasmodium berghei*-infected mice [10, 11]. However, the possible application of a combination of kaempferol with widely used artemisinin derivative, artesunate, in combating malaria has not yet been investigated. Therefore, this study was aimed to investigate the therapeutic effect of a combination of kaempferol and artesunate in *P. berghei*-infected mice using *in vivo* model.

## 2. Materials and Methods

### 2.1. Drugs and Reagents

Artesunate (ART), kaempferol (KMF), and Tween-80 were obtained from Sigma-Aldrich (St. Louis, MO, USA). All reagents were of analytical grade. The selected doses of ART and KMF were prepared in 20% Tween-80 and administered orally by gavage. The untreated group was given 20% Tween-80 only.

### 2.2. Experimental Mice

ICR male mice, 4–6 weeks old, weighing 25–30 g at the time of primary infection were used throughout this study. They were obtained from the National Laboratory Animal Center, Mahidol University, Thailand, and kept in an animal room with temperatures of 22–25°C, 12 h light-dark cycle. The animals were allowed free access *ad libitum* to a pellet diet and clean water. All experiments involving experimental mice were approved by the Animal Ethical Committee of Walailak University (AE002/2019).

### 2.3. Rodent Malaria Parasite

In this study, chloroquine-sensitive *Plasmodium berghei* strain ANKA (PbANKA) obtained from Malaria Research and Reference Reagent Resource Center (MR4, https://www.beiresources.org/About/MR4.aspx) was used. The parasite was maintained by intraperitoneal (IP) injection of 1 × 10^7^ parasitized erythrocytes of PbANKA into naïve ICR mice. Propagation of parasite was monitored by microscopy of Wright-Giemsa stained blood film, and percent parasitemia was subsequently calculated using

(1) $$\begin{array}{c} \%\;\mathrm{parasitemia}=\frac{\text{number of parasitized erythrocytes}}{\text{number of total erythrocytes}}\times 100. \end{array}$$

### 2.4. Antimalarial Suppressive Test

The ED_50_ of ART and KMF was estimated using the standard 4-day suppressive test as previously described [12]. Forty-five naïve ICR mice were infected with 1 × 10^7^ parasitized erythrocytes of PbANKA by IP injection. After 2 h, the mice were divided into 9 groups of 5 mice each. The test groups were given KMF (1, 5, 20, and 100 mg/kg) and ART (1, 3, 10, and 30 mg/kg) orally. The untreated group received 10 ml/kg of 20% Tween-80. The drugs were treated once a day for 4-consecutive days (D0–D3). At D4, parasitemia was estimated using microscopy of Wright-Giemsa stained thin blood film, and percent parasite suppression was subsequently calculated using

(2) $$\begin{array}{c} \%\text{ inhibition}=\frac{\left(\text{parasitemia of untreated group}-\text{parasitemia of treated group}\right)}{\text{parasitemia of untreated group}}\times 100. \end{array}$$

### 2.5. *In Vivo* Antimalarial Combination Test

In order to obtain the combination potency of the ART and KMF, antimalarial activity was assayed using the standard 4-days suppressive test previously described above. Twenty naïve ICR mice were administered by IP infection with 1 × 10^7^ parasitized erythrocytes of PbANKA. After 2 h, the mice were divided into 5 groups of 5 mice each and given the combination drugs at the doses of their respective ED_50_ and fixed-ratio combination (1 : 1) of their respective ED_50_ 1/2, 1/4, and 1/8. The untreated group was given 10 ml/kg of 20% Tween-80. The treatment was carried out once a day for 4-consecutive days (D0–D3). Parasitemia and percent suppression of parasite growth were subsequently calculated on D4.

### 2.6. Determination of Mean Survival Time

The mortality of all experimental mice was daily monitored, and the number of days from parasite inoculation up to death was recorded throughout the follow-up period. The mean survival time (MST) was calculated using the following formula [13]:

(3) $$\begin{array}{c} \text{MST}=\frac{\text{sum of survival time of all mice in a group}}{\text{total number of mice in that group}}. \end{array}$$

### 2.7. Statistics

All results were expressed as the mean ± standard error of the mean (SEM) using GraphPad Prism (GraphPad Software version 5.01, Inc., USA). The nonlinear regression function for the sigmoidal dose-response variable slope was used to calculate the best-fit ED_50_. One-way ANOVA followed by Tukey's post hoc test was used to compare the mean of measured parameters. The *P* < 0.05 at 95% confidence was regarded as statistically significant. Moreover, a combination index (CI), which defines synergism (CI < 1), additive effect (CI = 1), and antagonism (CI > 1), was automatically simulated by CompuSyn (CompuSyn software, ComboSyn, Inc., USA) [14].

## 3. Results

### 3.1. Antimalarial Activity of KMF and ART against PbANKA-Infected Mice

**Figure 1:**
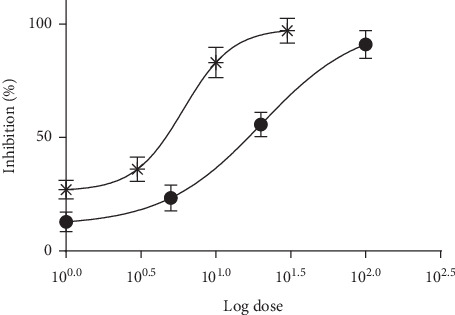
Dose-response curve of antimalarial activity of (^*∗*^) ART and (•) KMF against PbANKA-infected mice.

The chemosuppressive antimalarial activity of KMF and ART against PbANKA-infected mice is shown in Figure 1. KMF presented a significant (*P* < 0.05) dose-dependent inhibition in parasitemia levels with similar inhibition as in ART-treated groups. From the dose-response curve, the ED_50_ of KMF and ART was 20.06 ± 2.65 and 6.06 ± 1.33 mg/kg, respectively.

### 3.2. Combination of KMF and ART against PbANKA-Infected Mice

**Figure 2:**
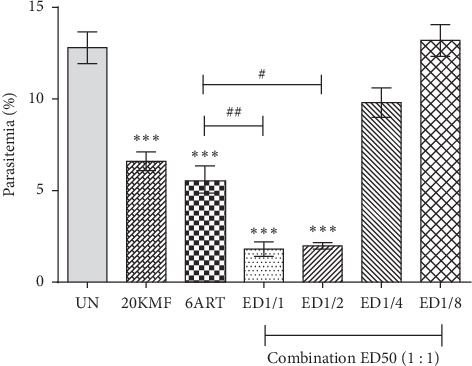
Antimalarial activity of combination treatment against PbANKA-infected mice. Groups of ICR mice (5 mice of each) were inoculated with 1 × 10^7^ parasitized erythrocytes of PbANKA by IP injection. They were then given the combination drugs of ART and KMF at the doses of their respective ED50 and fixed-ratio combination (1 : 1) of their respective ED50 of 1/2, 1/4, and 1/8. The treatment was carried out once a day for 4-consecutive days (D0–D3). Parasitemia was subsequently estimated on D4. ^^*∗∗∗*^^*P* < 0.001, compared to the untreated group. ^#^*P* < 0.05 and ^##^*P* < 0.01, compared to 6ART. UN: untreated group; 20KMF: 20 mg/kg of kaempferol; 6ART: 6 mg/kg of artesunate. Results were shown as mean ± SEM.

The combination of KMF and ART at the doses of their respective ED_50_ and fixed-ratio combination (1 : 1) of their respective ED_50_ of 1/2 exerted a significant (*P* < 0.001) reduction of parasitemia, compared to the untreated group with the CI values of 0.86 and 0.47, respectively, indicating synergism (Figure 2 and Table 1). Additionally, significant (*P* < 0.05) inhibition was also observed in these combinations when compared with 6 mg/kg of the ART-treated group. However, the combination of KMF and ART at the doses of their respective at 1/4 ED_50_ and 1/8 ED_50_ did not show the significant inhibition as compared to untreated and 6 mg/kg of ART-treated groups with the CI values of 4.64041 and 85.9454, respectively, indicating antagonism (Table 1).

### 3.3. Effect of KMF and ART on MST

From the 4-day suppressive test, 5 (14.4 ± 1.9 days), 20 (22.8 ± 1.9 days), and 100 (33.6 ± 4.0 days) mg/kg of KMF produced significantly (*P* < 0.05) prolonged MST in a dose-dependent manner compared to the untreated group (8.2 ± 1.9 days). Moreover, combination drugs at the doses of their respective ED_50_ (51.8 ± 2.2 days) and fixed-ratio combination (1 : 1) of their respective at 1/2 ED_50_ (43.0 ± 2.1 days) showed significant (*P* < 0.001) prolonged MST when compared to untreated (8.2 ± 1.9 days) and 10 mg/kg of ART- (28.4 ± 2.9 days) treated groups. However, the MST was not significantly prolonged in the combination of the respective at 1/4 ED_50_ (9.4 ± 1.7 days) and 1/8 ED_50_ (9.0 ± 1.6 days) (Table 2).

## 4. Discussion

ACT is now the major strategy for malarial treatment due to the high efficacy and low probability of antimalarial drug resistance development [15]. This study focused on the combination of KMF and ART, with the aim of developing a new ACT. Using the chronic toxicity test, KMF in mice has been previously reported to be safe at the dose up to 2,000 mg/kg in mice [10]. Moreover, the susceptibility test in the suppressive model of KMF against PbANKA showed potent antimalarial activity in a dose-dependent manner. Accordingly, this was a basis for the selection of the doses (1, 5, 10, 20, and 100 mg/kg) of KMF for the present ED_50_ evaluation. KMF exerted a significant (*P* < 0.05) dose-dependent antimalarial activity with similar inhibition as in ART-treated groups. It was due to the fact that antioxidant and anticancer activities of KMF might be responsible for its antimalarial activity [7, 10, 11]. For our finding, the present study reports for the first time ED_50_ level of KMF in the antimalarial *in vivo* model.

The combination treatment (1 : 1) of ED_50_ values of KMF and ART produced a more significant inhibition in parasitemia compared to the use of only ART. The synergistic effect was pronounced when the KMF and ART were used together at the doses of 1/2 ED_50_ each. This may be related to a rapid onset of antimalarial activity of KMF in combination with ART. Unfortunately, the mode of action involved in the enhanced antimalarial activity obtained with KMF and ART combinations has not yet been investigated. Further studies should be performed to highlight the mechanisms behind its antimalarial interactions. Additionally, the combination of KMF and ART at the doses of ED_50_ 1/4 and ED_50_ 1/8 displayed antagonistic effect. This could be due to the subtherapeutic doses used.

It has been described that the tested compound that could significantly prolong the MST of infected mice is considered as active [13]. For the result of MST obtained from this study, PbANKA-infected mice treated with KMF, ART, and its combination had significantly longer MST than the untreated group. This might be due to the antimalarial activity of these compounds.

## 5. Conclusion

From this study, it can be concluded that the combination at a fixed ratio (1 : 1) of KMF and ART exerted potent antimalarial activity with a synergistic effect against PbANKA-infected mice. In addition, significant prolonged MST in PbANKA-infected mice treated with these drugs was also observed. Accordingly, KMF could serve as a potential partner drug that can be combined with ART for the treatment of malarial infection.

## Figures and Tables

**Table 1:** Combination index values of the combination of artesunate and kaempferol against *Plasmodium berghei* ANKA-infected mice.

Test	Dose (mg/kg)			CI value
	KMF	ART		
Combination (1 : 1)	ED_50_	20	6	0.86154^a^
	ED_50_ 1/2	10	3	0.46680^a^
	ED_50_ 1/4	5	1.5	4.64041^b^
	ED_50_ 1/8	2.5	0.75	85.9454^b^

^a^CI < 1; synergism, ^b^CI > 1; antagonism.

**Table 2:** Effect of combination treatment between artesunate and kaempferol on mean survival time of *Plasmodium berghei* ANKA-infected mice.

Test	Dose	MST (days)
Untreated	10 ml/kg	8.2 ± 1.9

KMF	1 mg/kg	10.2 ± 1.3
	5 mg/kg	14.4 ± 1.9^*∗*^
	20 mg/kg	22.8 ± 1.9^*∗∗∗*^
	100 mg/kg	33.6 ± 4.0^*∗∗∗*^

ART	1 mg/kg	14.6 ± 1.8^*∗*^
	3 mg/kg	17.6 ± 2.1^*∗∗∗*^
	10 mg/kg	28.4 ± 2.9^*∗∗∗*^
	30 mg/kg	51.2 ± 4.1^*∗∗∗*^

Combination (1 : 1)	ED_50_ (20KMF + 6ART)	51.8 ± 2.2^*∗∗∗*^^,a^
	ED_50_ 1/2 (10KMF + 3ART)	43.0 ± 2.1^*∗∗∗*^^,a^
	ED_50_ 1/4 (5KMF + 1.5ART)	9.4 ± 1.7
	ED_50_ 1/8 (2.5KMF + 0.75ART)	9.0 ± 1.6

^*∗∗∗*^ *P* < 0.001, compared to the untreated group. ^a^*P* < 0.001, compared to 10 mg/kg of ART.

## Data Availability

The graphs or figures data used to support the findings of this study have been deposited in the Figshare repository (DOI: 10.6084/m9.figshare.7973849).
